# Risk Score for Predicting Dysphagia in Patients After Neurosurgery: A Prospective Observational Trial

**DOI:** 10.3389/fneur.2021.605687

**Published:** 2021-05-11

**Authors:** Li Zeng, Yu Song, Yan Dong, Qian Wu, Lu Zhang, Lei Yu, Liang Gao, Yan Shi

**Affiliations:** ^1^Neurosurgical Intensive Care Unit, Shanghai Tenth People's Hospital, School of Medicine, Tongji University, Shanghai, China; ^2^Department of Nursing, Shanghai Tenth People's Hospital, School of Medicine, Tongji University, Shanghai, China; ^3^Department of Neurosurgery, Shanghai Tenth People's Hospital, School of Medicine, Tongji University, Shanghai, China; ^4^Clinical Medicine Scientifific and Technical Innovation Park, Shanghai Tenth People's Hospital, Shanghai, China; ^5^Department of Anesthesiology, Dongfang Hospital Affifiliated to Tongji University, Shanghai, China

**Keywords:** prediction, neurointensive care unit, nomogram, neurosurgery, dsyphagia

## Abstract

**Background:** Acquired dysphagia is common in patients with tracheal intubation and neurological disease, leading to increased mortality. This study aimed to ascertain the risk factors and develop a prediction model for acquired dysphagia in patients after neurosurgery.

**Methods:** A multicenter prospective observational study was performed on 293 patients who underwent neurosurgery. A standardized swallowing assessment was performed bedside within 24 h of extubation, and logistic regression analysis with a best subset selection strategy was performed to select predictors. A nomogram model was then established and verified.

**Results:** The incidence of acquired dysphagia in our study was 23.2% (68/293). Among the variables, days of neurointensive care unit (NICU) stay [odds ratio (OR), 1.433; 95% confidence interval (CI), 1.141–1.882; *P* = 0.005], tracheal intubation duration (OR, 1.021; CI, 1.001–1.062; *P* = 0.175), use of a nasogastric feeding tube (OR, 9.131; CI, 1.364–62.289; *P* = 0.021), and Acute Physiology and Chronic Health Evaluation (APACHE)-II C score (OR, 1.709; CI, 1.421–2.148; *P* < 0.001) were selected as risk predictors for dysphagia and included in the nomogram model. The area under the receiver operating characteristic curve was 0.980 (CI, 0.965–0.996) in the training set and 0.971 (0.937–1) in the validation set, with Brier scores of 0.045 and 0.056, respectively.

**Conclusion:** Patients who stay longer in the NICU, have a longer duration of tracheal intubation, require a nasogastric feeding tube, and have higher APACHE-II C scores after neurosurgery are likely to develop dysphagia. This developed model is a convenient and efficient tool for predicting the development of dysphagia.

## Introduction

Post-extubation dysphagia is a common complication in mixed intensive care units (ICUs), causing aspiration pneumonia, malnutrition, and dehydration, and increasing the length of hospital stay and mortality (1–5). The prevalence of post-extubation dysphagia ranges from 3 to 62%, which leads to increased healthcare-related costs (6, 7). The condition is even worse in patients with neurological diseases. In addition to neuromuscular disease, acquired neurological disease is also a high risk for dysphagia. According to reports, 28–65% of patients with acute stroke experience difficulty in swallowing (8), same as brain tumors (9). Even in patients with a non-traumatic subarachnoid hemorrhage, the incidence of dysphagia is 16.33% (10). In fact, previous clinical studies have identified neurological diseases as a significant risk factor for the development of dysphagia (11, 12).

Therefore, early and timely assessment of whether the patient has dysphagia can significantly reduce the occurrence of pneumonia and further reduce mortality (13). Although dysphagia has a high incidence rate in patients with neurological diseases and is an important predictor of poor prognosis, few relevant predictive studies have been conducted over the past few decades. A nomogram is a graphical statistical device that makes it possible to qualify individual prediction probability based on patients' characteristics. Currently, nomograms have been widely used in the medical field, helping to guide clinical decision-making.

In the present study, we performed a multicenter prospective observational study to identify risk factors for acquired dysphagia in patients after neurosurgery. Additionally, we developed and validated a simple and reliable model for predicting dysphagia in patients after neurosurgery.

## Methods

### Study Design and Participants

A multicenter prospective observational study was performed in the neurointensive care unit (NICU) of three tertiary care teaching hospitals. The prerequisites for extubation in patients after undergoing neurosurgery included having spontaneous breathing, s‘ hemodynamics, and presumably protecting the airway (positive cough reflex, less tracheobronchial secretions, and normal consciousness level). Eligible patients were consecutively included in our study between May 2018 and December 2019. Inclusion criteria were as follows: (1) age >18 years; (2) admitted to the NICU after neurosurgery. Exclusion criteria were as follows: (1) patients with primary laryngopharyngeal diseases, laryngopharyngeal mass, or any other situation leading to dysphagia before enrollment; (2) patients who could not be extubated or with tracheotomy; (3) patients who rejected to participated in standardized swallowing assessment (SSA) for any reason; and (4) patients who failed to finish the first SSA (e.g., Some patients were not allowed to drink or eat due to their condition) or failed to complete all the follow up (e.g., Some patients were thought that the SSA was cumbersome and they were unwilling to participate again after the first test). This study was approved by the Ethics Committees of Shanghai Tenth People's Hospital, affiliated with Tongji University (Shanghai, China).

### Data Collection

#### Questionnaire Design and Distribution

**Figure 1:**
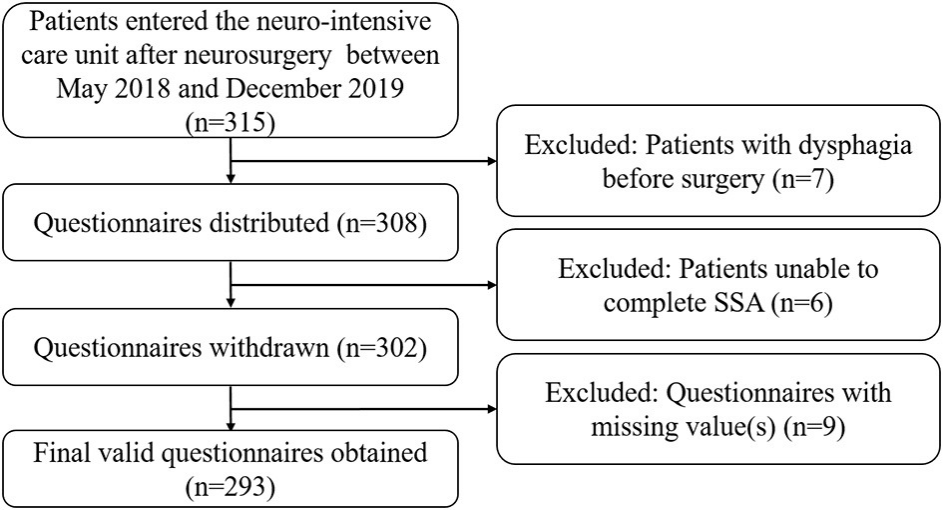
Flowchart of the study design. NICU, neurointensive care unit; SSA, standardized swallowing assessment.

Data collection was performed using a self-designed questionnaire (Supplementry Materials), which mainly comprised three sections: patients' demography, past medical history, and clinical features. The nurses in our research team have strict criteria: (a) work for at least 5 years; (b) with a master's degree; (c) training and testing of SSA by one nurse manager. Questionnaires were distributed to qualified nurses in the NICU. All eligible patients underwent systematic bedside screening and filled out the questionnaires, which were then reviewed by a neurologist. Figure 1 shows a flowchart of the experimental design.

#### Parameters Acquirement

After collecting all the questionnaires, data were extracted. Demographic features, past medical history, and clinical features were collected. Dysphagia was evaluated by SSA within 24 h of extubation, as previously reported (14) (Supplementary Materials). Briefly, the SSA scale comprised three parts, including clinical examination, a stage 1 water swallow test (WST), and a stage 2 WST. Patients with an abnormal consciousness level, breath pattern, lip closure, palate movement, laryngeal function, gag reflex, voluntary cough, or loss of control of the head and trunk were valued as dysphagia, and SSA was terminated. In stage 1 WST, the patient was given 5 ml of water three times consecutively. If the patient finished stage 1 WST two out of three times without dribbling water, laryngeal movements on the attempted swallow, “repeated movements” felt, coughing, stridor, or any abnormal laryngeal function after swallowing, they then proceeded to the stage 2 WST. In stage 2 WST, the patient was instructed to drink 60 ml of water. If the patient did not display coughing, stridor, aspiration, or any abnormal laryngeal function after swallowing, the patient was considered not having dysphagia. In the present study, patients had normal consciousness levels and normal breathing patterns at the first evaluation (the prerequisites for extubation). Patients were screened every 8 h until dysphagia was diagnosed or they were discharged from the hospital. The baseline severity of the disease was assessed using Acute Physiology and Chronic Health Evaluation (APACHE)-II scores at admission, and each item was listed separately. The Richmond Agitation-Sedation Scale (RASS) was used to assess the sedation state of patients when assessing SSA. Days in the NICU, hours on mechanical ventilation, hours with tracheal intubation, and hours with sedation relaxant usage were recorded. Questionnaires with missing values were discarded.

### Statistical Analyses

All data were randomly divided into a training set and a validation set at a ratio of 7:3. The continuous variables were checked for normal distribution using a Shapiro-Wilk test. Data are expressed as medians and interquartile ranges for continuous variables and *n* (%) for categorical variables. Univariate analysis between the two groups in terms of categorical variables and continuous variables was conducted using a chi-squared test or Mann–Whitney *U*-test, respectively. The variance of inflation factors (VIF) was calculated to determine multicollinearity. Factors with VIF >10 were considered as serious collinearity, were excluded from further analyses. A logistic regression analysis was then performed to identify the risk factors for predicting dysphagia. Only variables with *P* < 0.05 in the univariate analysis were included in the next analysis. The best subset selection strategy was employed to construct a logistic regression model, and the Bayesian Information Criteria (BIC) was used to select the best model from all possible subsets. Then, a nomogram was developed based on the results of the logistic regression, which was validated using the validation set. The model was also internally validated using all the data by performing 10-fold cross-validation. The area under the receiver operating characteristic curve (AUC) was calculated to measure the discrimination performance. The calibration curve was used to test the reliability with bootstraps of 1,000 resamples, which described the degree of fit between the actual and nomogram-predicted probability. The Brier score was used to measure the accuracy of probabilistic predictions, which ranged from 0 to 1. The lower the Brier score, the better the predictions of dysphagia were calibrated. Decision curve analysis was used to evaluate the model's profitability. All statistical analyses were performed using R statistical software (version 4.0.0; R Foundation for Statistical Computing, Vienna, Austria).

## Results

### Demographic and Clinical Characteristics

After screening according to our exclusion criteria, we collected 293 valid questionnaires in this study. In total, 206 patients (48 patients with dysphagia, 23.3%) were included in the training set, while 87 patients (20 patients with dysphagia, 23.0%) were included in the validation set.

The demographics and clinical characteristics of patients with and without dysphagia are shown in Table 1. Considering that the APACHE-II score includes patients' age, we listed the four items separately. Among all the variables, age, diagnostic category, hypertension, NICU stay, mechanical ventilation, protective restraint, nasogastric feeding tube, tracheal intubation, sedation, relaxants, muscle strength grade, RASS score, and APACHE II score were significantly different between the two groups.

**Table 1:** Baseline characteristics comparison between patients with dysphagia or not.

**Variables**	**Overall**	**Dysphagia screening negative**	**Dysphagia screening positive**	***p*-value**
	***N* = 293**	***N* = 225**	***N* = 68**	
Age, years	53.0 [44.0, 63.0]	51.0 [42.0, 62.0]	60.5 [53.0, 66.0]	<0.001
Male, *n* (%)	151 (51.5)	118 (52.4)	33 (48.5)	0.669
Height, cm	165.0 [160.0, 170.0]	165.0 [160.0, 171.0]	162.5 [158.0, 170.0]	0.074
Weight, kg	68.0 [60.0, 75.0]	68.0 [60.0, 75.0]	68.0 [60.0, 75.0]	0.813
BMI	24.5 [22.0, 26.6]	24.5 [21.9, 26.6]	24.4 [22.0, 27.0]	0.577
Diagnostic Category (%)				0.001
Neurovascular disease	132 (45.1)	105 (46.7)	27 (39.7)	
Central nervous system tumor	146 (49.8)	115 (51.1)	31 (45.6)	
Traumatic brain injury	12 (4.1)	4 (1.8)	8 (11.8)	
Others	3 (1.0)	1 (0.4)	2 (2.9)	
**Past medical history**				
Diabetes mellitus, *n* (%)	31 (10.6)	23 (10.2)	8 (11.8)	0.891
Hypertension, *n* (%)	88 (30.0)	52 (23.1)	36 (52.9)	<0.001
Heart failure, *n* (%)	48 (16.4)	34 (15.1)	14 (20.6)	0.378
Arrhythmia, *n* (%)	30 (10.2)	24 (10.7)	6 (8.8)	0.833
Chronic renal failure, *n* (%)	19 (6.5)	15 (6.7)	4 (5.9)	1
Previous stroke, *n* (%)	174 (59.4)	141 (62.7)	33 (48.5)	0.052
**Clinical features**				
NICU stay, days	2.0 [1.0, 3.0]	2.0 [1.0, 2.0]	6.0 [3.0, 10.0]	<0.001
Mechanical ventilation, hours	4.0 [3.0, 5.0]	3.0 [2.5, 5.0]	5.0 [3.2, 6.0]	<0.001
Protective restraint, *n* (%)	9 (3.1)	1 (0.4)	8 (11.8)	<0.001
Nasogastric feeding tube, *n* (%)	47 (16.0)	9 (4.0)	38 (55.9)	<0.001
Tracheal intubation type (%)				0.171
6.5	84 (28.7)	62 (27.6)	22 (32.4)	
7.0	113 (38.6)	94 (41.8)	19 (27.9)	
7.5	38 (13.0)	29 (12.9)	9 (13.2)	
8.0	58 (19.8)	40 (17.8)	18 (26.5)	
Tracheal intubation duration, hours	4.0 [3.0, 7.0]	3.0 [2.5, 5.0]	29.5 [7.8, 95.8]	<0.001
Sedation, hours	3.5 [3.0, 5.0]	3.0 [2.3, 5.0]	5.0 [3.8, 7.0]	<0.001
Relaxants, hours	4.0 [3.0, 6.0]	3.0 [2.0, 5.0]	6.0 [4.0, 8.0]	<0.001
Muscle strength grade III and IV, *n* (%)	44 (15.0)	7 (3.1)	37 (54.4)	<0.001
RASS score	0.0 [0.0, 0.0]	0.0 [0.0, 0.0]	0.0 [-1.0, 0.0]	<0.001
APACHE-II score	20.0 [19.0, 21.0]	20.0 [19.0, 21.0]	23.0 [21.0, 29.2]	<0.001
APACHE-IIA	2.0 [0.0, 3.0]	2.0 [0.0, 3.0]	3.0 [2.0, 5.0]	<0.001
APACHE-IIB	2.0 [2.0, 2.0]	2.0 [2.0, 2.0]	2.0 [2.0, 5.0]	<0.001
APACHE-IIC	0.0 [0.0, 1.0]	0.0 [0.0, 0.0]	6.0 [2.0, 8.0]	<0.001
APACHE-IID	15.0 [13.0, 17.0]	15.0 [14.0, 17.0]	13.5 [9.0, 17.0]	0.001

*NICU, neurointensive care unit; BMI, body mass index; RASS, richmond agitation-sedation scale; APACHE, acute physiology and chronic health evaluation. Continuous variables present with median and inter-quartile range; Categorical variables present with frequencies*.

### Risk Factors of DG

All variables were included in the multivariate logistic regression analysis. The best subset was chosen based on the smallest BIC. Days of NICU stay, tracheal intubation duration, the existence of a nasogastric feeding tube, and APACHE-II C score were selected as risk predictors for dysphagia. No variables were meeting the criteria for multicollinearity (VIF >10). The results of multivariable analyses for dysphagia in the training set are shown in Table 2.

**Table 2:** Model for prediction of dysphagia.

**Variables**	**Coefficient**	**OR (CI)**	***p*-value**
NICU stay, days	0.360	1.433 (1.141–1.882)	0.005
Tracheal intubation duration, hours	0.021	1.021 (1.001–1.062)	0.175
Nasogastric feeding tube, *n* (%)	2.212	9.131 (1.364–62.289)	0.021
APACHE-II C	0.536	1.709 (1.421–2.148)	<0.001

*NICU, neurointensive care unit; APACHE, acute physiology and chronic health evaluation; OR, odds ratio*.

### Establishment and Verification of a Nomogram for DG

**Figure 2:**
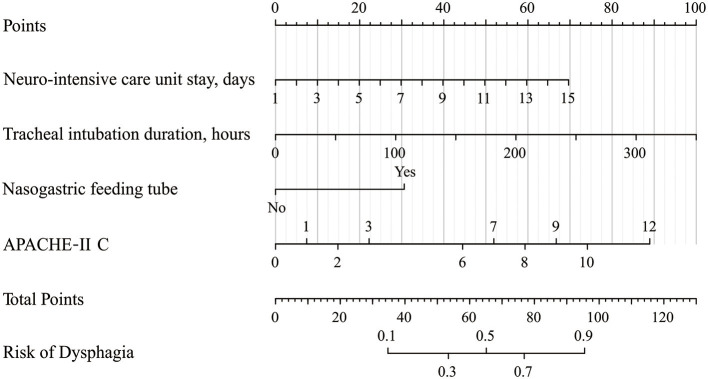
Nomogram for predicting the risk of dysphagia. Points are assigned for NICU stay, tracheal intubation duration, nasogastric feeding tube, and APACHE-II C score. The “Points” displays prognostic points. The “total points” are calculated as the sum of the individual score of each variable, which is then used to find the appropriate position on the “Risk of Dysphagia” axis to determine the patient's individual risk of dysphagia. For example, if one patient now stayed in NICU for 7 days (about 30 points), the tracheal intubation lasted for 35 h (about 10 points), without the nasogastric feeding tube (0 points), the APACHE-II C score was 2 at admission (about 15 points), the total points for this patient were 55 points. The risk of dysphagia for this patient was about 33%. NICU, neurointensive care unit; APACHE-II, Acute Physiology and Chronic Health Evaluation.

According to the results of multivariate logistic regression analysis, four variables, including days of NICU stay, tracheal intubation duration, the existence of a nasogastric feeding tube, and APACHE-II C score were ultimately chosen as predictors to develop a nomogram model (Figure 2). In this model, tracheal intubation duration showed the greatest effect on dysphagia, followed by APACHE-II C score and then days of NICU stay. The existence of a nasogastric feeding tube was the smallest risk factor for dysphagia. The risk of dysphagia for patients after neurosurgery was found to be positively correlated with the sum of the four predictors' points in the nomogram model.

**Figure 3:**
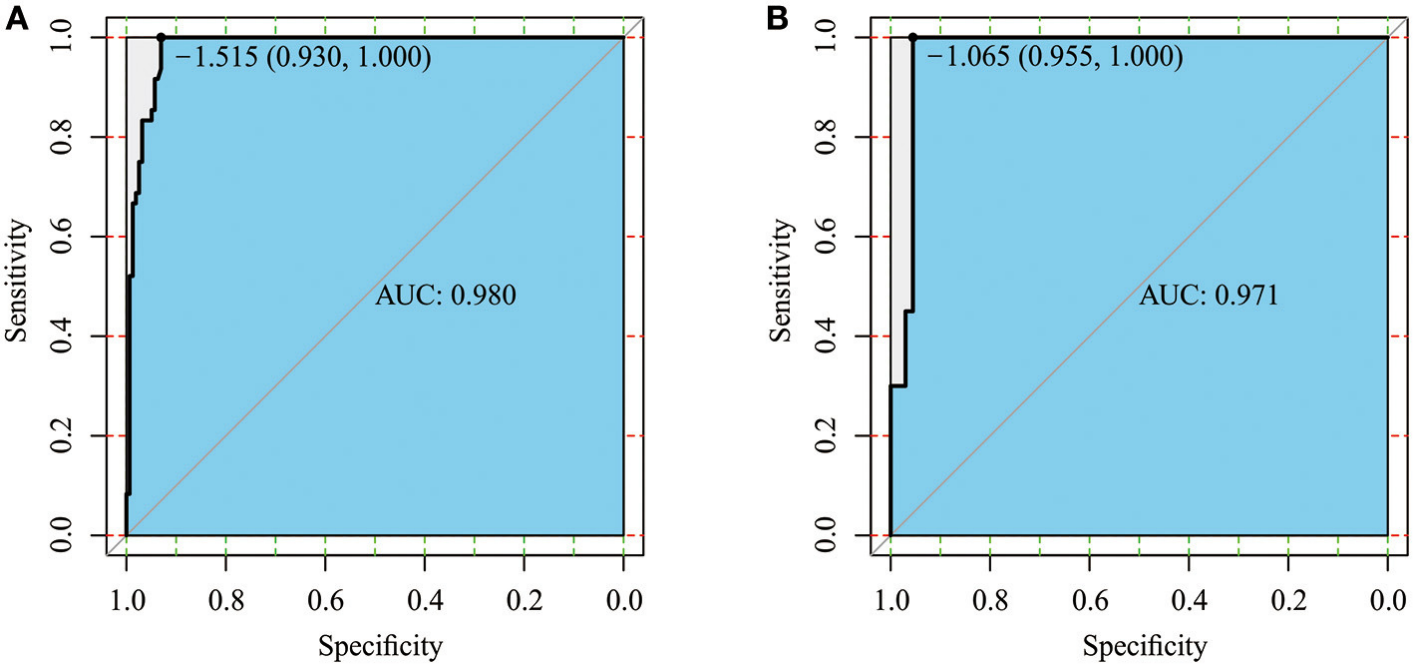
Receiver operating characteristic (ROC) curve of the training set **(A)** and validation set **(B)**.

AUC was used to assess the discriminative performance of the nomogram model in the training set [0.980, 95% confidence interval (CI): 0.965–0.996, Figure 3A] and externally validated in the validation set (0.971, 95% CI: 0.937–1, Figure 3B). Using 10-fold cross-validation in the total data, we further evaluated the predictive accuracy of the model. The max AUC was 0.994 (95% CI: 0.977–1).

**Figure 4:**
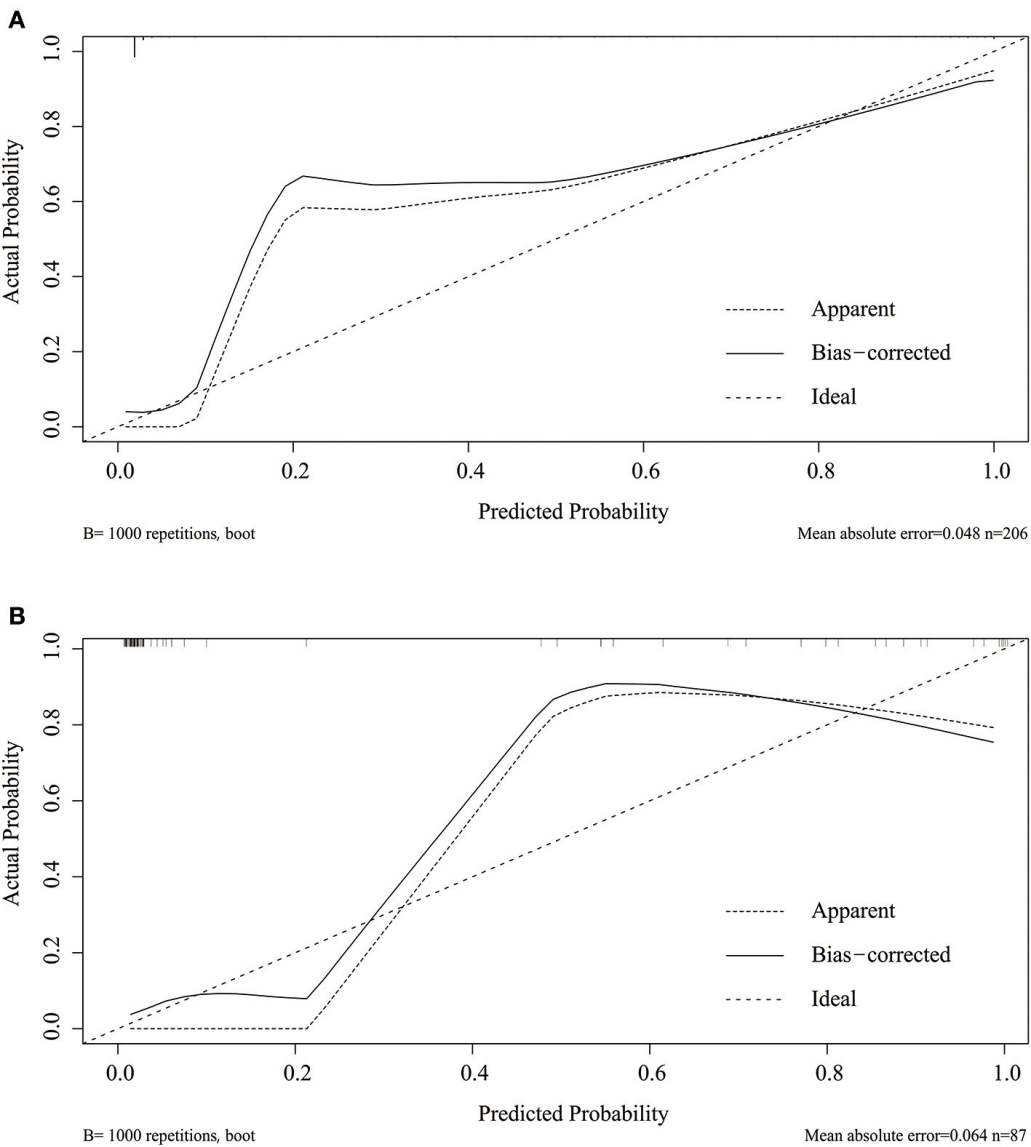
Calibration plot of the nomogram in the training **(A)** and validation set **(B)**. Predictions generated from the model are plotted against actual patient outcomes. The 45-degree line represents the perfect model calibration. The dotted line (apparent) indicates calibration when the model is applied to each set, and the solid line (bias-corrected) indicates calibration when the model is applied to the bootstrap set.

**Figure 5:**
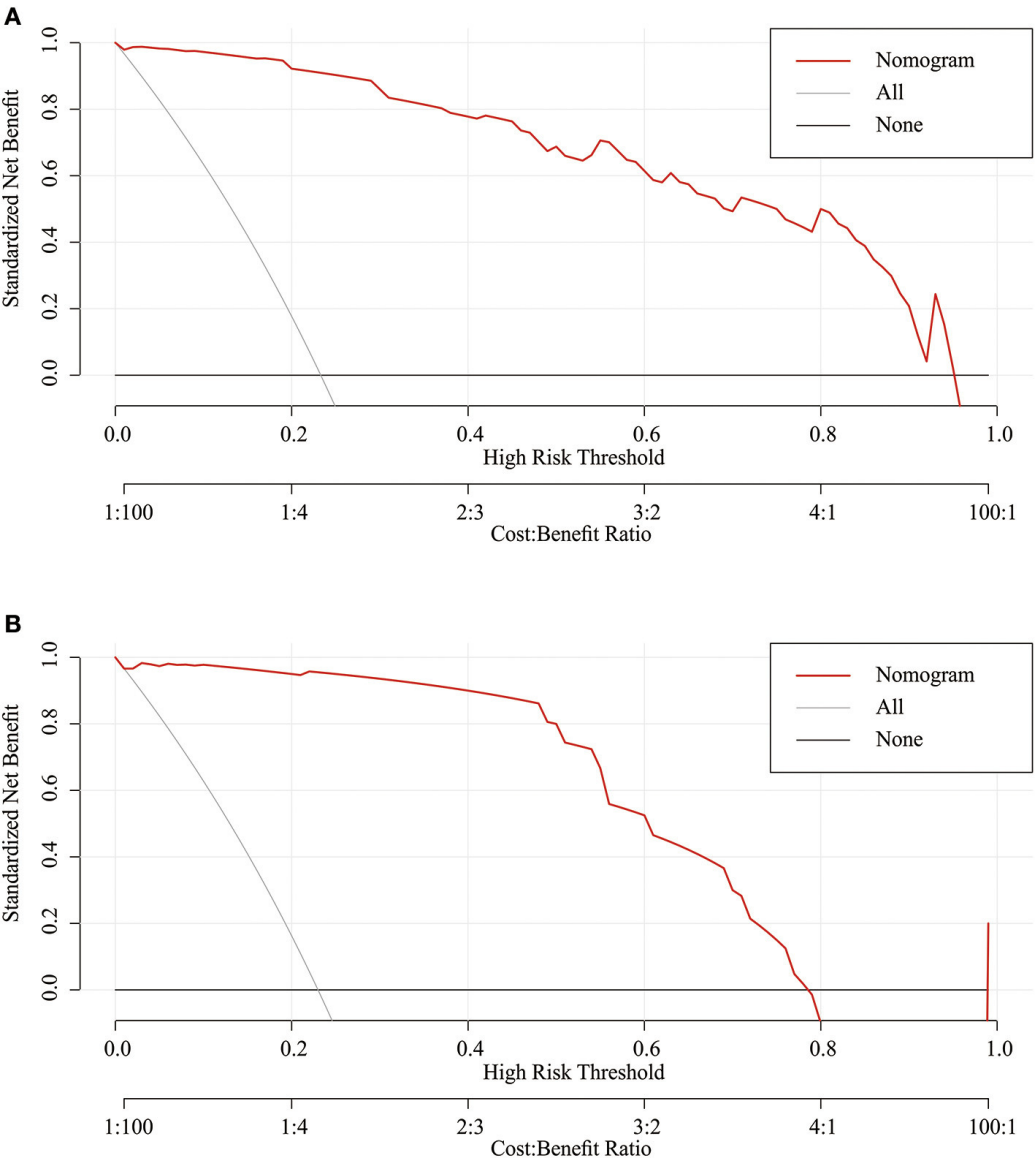
Decision curve analysis of the nomogram in the training set **(A)** and validation set **(B)**. The red line displays the net benefit of our model. The gray line assumes that all patients develop dysphagia. The black line assumes that no patients develop dysphagia.

Considering that discrimination alone was insufficient to assess the prediction capability of the model, we verified the performance of calibration using a calibration plot and the Brier score. A calibration plot with 1,000 bootstraps suggested good prediction performance in both the training set (Figure 4A) and validation set (Figure 4B), with mean absolute errors of 0.048 and 0.057, respectively. The Brier score can quantify the calibration performance. The Brier scores in the two sets were 0.045 and 0.056, respectively. A decision curve analysis was developed to evaluate the prognostic value of the prediction model. Figure 5 shows the decision curve analysis of the two sets. This indicates that patients can benefit from our nomogram model.

## Discussion

In the present study, we performed a multicenter prospective observational study in the NICU. Days of NICU stay, tracheal intubation duration, the existence of a nasogastric feeding tube, and APACHE-II C score were identified as risk factors for dysphagia in patients after neurosurgery. Using these four variables, we developed a nomogram model to calculate the risk of dysphagia in these patients.

Tracheal intubation and neurological diseases are the two main risk factors for acquired dysphagia (6, 8). Tracheal intubation often causes mechanical injury, such as mucosal abrasion, laryngeal edema, or a decrease in laryngeal sensation. Prolonged intubation can lead to tongue, pharynx, and larynx muscles disuse atrophy (15, 16). Patients with a neurological disease or critical illness often have damage to the cortex and subcortical structures, which decreases the coordination of the swallowing reflex (8, 17, 18). Acute phase admission is also a crucial factor for dysphagia (11, 12), concentrating on the main diseases with less attention to nutrition and activity (19). However, there are few studies on patients after neurosurgery, which contains all three factors. In our study cohort, the incidence of dysphagia was 23.2% (68/293), which is relatively low compared with other studies (6, 7). The reason why there were fewer positive patients may be due to fewer emergency patients (47/293 patients with APACHE-II B score of 5) and a higher baseline APACHE-II C score (the median of the patient's APACHE II C score is 0) in our institutes.

The SSA was first proposed by Smithard et al. in 1996 (14), and also validated by other patients with neurological diseases (20). In the meantime, it's easy to perform at the bedside than videofluoroscopy or fibreoptic endoscopy and safer than Kubota Water Swallowing Test. It is a good tool for screening dysphagia at the bedside (20, 21). However, it still requires a well-trained nurse and is time-intensive. Our prediction model only included four factors; however, it demonstrated excellent discrimination and calibration. Using our nomogram tool, clinicians or nurses can easily perform bedside screening in the NICU to screen patients who are at high risk for acquired dysphagia. Patients with a high score should further undergo a systematic examination and measures should be taken to prevent the complication of acquired dysphagia. Aspiration pneumonia is a serious consequence of dysphagia, affecting 37–55% of patients with stroke (22). Screening and timely assessment of dysphagia could significantly decrease the risk of pneumonia. In fact, the incidence of pneumonia increases by 1% every day the evaluation is delayed (13). For people with acute stroke, dysphagia increases the risk of malnutrition by up to 12 times. Therefore, patients with a high risk of dysphagia should be assessed thoroughly for the risk of malnutrition, and enteral nutrition or parenteral nutrition should be considered (23).

These four variables are all routine and easy to obtain. A longer ICU stay has been previously proposed as a potential risk factor for post-extubation dysphagia (10), which was further confirmed in our study. The existence of a nasogastric feeding tube can cause mechanical damage to the pharyngeal mucosa, laryngopharyngeal edema, sensory deficits, and swallowing-related muscle atrophy, which decreases swallowing function (24). Several studies have also reported the risk of feeding tube exposure (11, 25). In addition, the long-term existence of the feeding tube can increase the risk of aspiration (26, 27). Although swallowing can be achieved without conscious input, cortical control plays a critical role in the swallowing process (18). The baseline APACHE-II C score was calculated using the Glasgow Coma Scale (GCS), which is used to evaluate the state of a patient's consciousness. Patients with decreased GCS are likely to have dysphagia and aspiration (10, 28).

Importantly, our primary goal was to build a prediction model. Therefore, we attempted to achieve the best discriminatory ability and calibration possible. So although tracheal intubation duration is not statistically significant (*p* > 0.05), it also stays in our final model. Tracheal intubation duration was previously reported as a risk factor for dysphagia (29). The tracheal tube can directly lead to trauma in normal anatomic structures and compress the laryngeal nerve, which leads to swallowing dysfunction (30).

Nevertheless, there are some limitations to our study. First, our study cohort was relatively small for prediction model development. Second, we did not include other previously reported risk factors in our model, such as advanced age (31–33), emergency admission (11, 12), and previous stroke (4, 18, 29, 34). Therefore, the discrimination and calibration of our model need to be further validated by an external cohort from other centers. Third, we didn't set the exclusion criteria for patients with pneumonia, obstructive sleep apnea, gastroesophageal reflux et al. However, these diseases share dysphagia as common sequelae, which may increase the positive rate of dysphagia. In the meantime, our questionnaire lacks information during the perioperative period. This could also bias our results. Lastly, there were relatively few patients positive for dysphagia in our study cohort, which may come from SSA. This screening tool cannot definitively rule out silent aspiration and laryngeal dysfunction. Few numbers of positive events may cause the model's prediction accuracy to be too high and thus not stable enough. In the future, more patients from different levels of medical institutions are needed to verify our model.

## Conclusions

Longer NICU stay, longer tracheal intubation duration, the existence of a nasogastric feeding tube, and higher APACHE-II C score were the main risk factors for acquired dysphagia in patients undergoing neurosurgery. Our model has good performance in terms of discrimination and calibration in predicting acquired dysphagia. It allows clinicians to screen patients with a high risk of dysphagia, thus providing them the ability to take timely preventative measures to reduce the complications associated with dysphagia.

## Data Availability Statement

The raw data supporting the conclusions of this article will be made available by the authors, without undue reservation.

## Ethics Statement

The studies involving human participants were reviewed and approved by Ethics Committee of Shanghai Tenth People's Hospital. The patients/participants provided their written informed consent to participate in this study.

## Author Contributions

LG and YSh: conception, design, and revising the article critically for intellectual content. LY and LZh: acquisition of data. LZe and YSo: analysis, interpretation of data, and drafting the article. LZe, YSo, YD, QW, LZh, LY, LG, and YSh: final approval of the version to be published and Agreement to be accountable for all aspects of the work in ensuring that questions related to the accuracy or integrity of any part of the work are appropriately investigated and resolved. All authors contributed to the article and approved the submitted version.

## Conflict of Interest

The authors declare that the research was conducted in the absence of any commercial or financial relationships that could be construed as a potential conflict of interest.
