# Supplementary material for: Risk Score for Predicting Dysphagia in Patients After Neurosurgery: A Prospective Observational Trial
Source: Front Neurol. 2021 May 11;12:605687. doi: 10.3389/fneur.2021.605687 (PMC8144441; doi:10.3389/fneur.2021.605687)
Supplement: Supplementary file 1 [file Table_1.docx]

#### **Questionnaire in patients after neurosurgery**

| Patient ID: | | Name: | | |
| --- | --- | --- | --- | --- |
| **(1) Demographic features** | | | | |
| Gender: | Age: | | | Height: |
| Weight | Body mass index: | | |  |
| **(2) Past medical history (Yes/No)** | | | | |
| Diabetes mellitus: | | | Hypertension: | |
| Heart failure: | | | Arrhythmia: | |
| Chronic renal failure: | | | Previous stroke: | |
| **(3) Clinical features (fill these blanks at admission)** | | | | |
| Diagnose: | | | | |
| APACHE-II score (total): | | | | |
| APACHE-IIA: | | APACHE-IIB: | | |
| APACHE-IIC: | | APACHE-IID: | | |
| **(4) Clinical features (fill these blanks at extubation)** | | | | |
| Mechanical ventilation (hours): | | | | |
| Tracheal intubation type (6.5/7.0/7.5/8.0): | | | | |
| Tracheal intubation duration (hours): | | | | |
| **(5) Clinical features (fill these blanks if the patients SSA positive or discharge)** | | | | |
| SSA (positive/negative): | | | | |
| Nasogastric feeding tube (yes/no): | | | | |
| Sedation (hours): | | | | |
| Relaxants (hours): | | | | |
| Muscle strength grade (0-V): | | | | |
| RASS score: | | | | |
| Protective restraint (yes/no): | | | | |
| NICU stay (days): | | | | |

#### **Bedside Swallowing Assessment (SSA)** (1)

| Patient ID: | Name: | | |
| --- | --- | --- | --- |
| **Clinical examination** | | | |
| Conscious level (Alert?) | Yes □ | / No □ |  |
| Head and trunk control (Normal?) | Yes □ | / No □ |  |
| Breathing pattern (Normal?) | Yes □ | / No □ |  |
| Lip closure (Normal?) | Yes □ | / No □ |  |
| Palate movement (Symmetrical?) | Yes □ | / No □ |  |
| Laryngeal function (Normal?) | Yes □ | / No □ |  |
| Gag (Present?) | Yes □ | / No □ |  |
| Voluntary cough (Normal?) | Yes □ | / No □ |  |
| **Stage 1: Give a teaspoon (5 mL** **) of water 3 times** **.** | | | |
| Dribbles water | None/once □ | / >once □ |  |
| Laryngeal movement on attempted swallow | Yes □ | / No □ |  |
| “Repeated movements” felt? | None/once □ | / >once □ |  |
| Cough on swallowing | None/once □ | / >once □ |  |
| Stridulous on swallowing | No □ | / Yes □ |  |
| Laryngeal function after swallowing (Normal?) | Yes □ | / No □ |  |
| **Stage 2: I** **f the swallow is normal in stage 1 (2 of 3 attempts)** **,** **try 60** **mL** **of water in a beaker.** | | | |
| Able to finish? | Yes □ | / No □ |  |
| Time taken to finish in seconds: |  |  |  |
| Number of sips: |  |  |  |
| Cough during or after swallowing | No □ | / Yes □ |  |
| Stridor during or after swallowing | No □ | / Yes □ |  |
| Laryngeal function after swallowing (Normal?) | Yes □ | / No □ |  |
| Do you feel aspiration is present? | No □ | / Yes □ |  |

| Clinical examination: (Normal/Abnormal) | Stage 1:  (Normal/Abnormal) | Stage 2:  (Normal/Abnormal) |
| --- | --- | --- |
| Dysphagia: | Positive □ | Negative □ |

Nurse name:

Doctor name: Date:

1. Smithard DG, O’Neill PA, Parks C, Morris J. Complications and outcome after acute stroke. Does dysphagia matter? Stroke. (1996) 27:1200–4. doi: 10.1161/01.STR.27.7.1200
